# Asymmetry and Heterogeneity: Part and Parcel in Cardiac Autonomic Innervation and Function

**DOI:** 10.3389/fphys.2021.665298

**Published:** 2021-09-16

**Authors:** Tjitske E. Zandstra, Robbert G. E. Notenboom, Jeroen Wink, Philippine Kiès, Hubert W. Vliegen, Anastasia D. Egorova, Martin J. Schalij, Marco C. De Ruiter, Monique R. M. Jongbloed

**Affiliations:** ^1^Department of Cardiology, Leiden University Medical Center, Leiden, Netherlands; ^2^Department of Anatomy and Embryology, Leiden University Medical Center, Leiden, Netherlands; ^3^Department of Anesthesiology, Leiden University Medical Center, Leiden, Netherlands

**Keywords:** autonomic nervous system, sympathetic cardiac nerves, vagal cardiac branches, vagus nerve, cardiac autonomic function, regional differences, anatomical sidedness, asymmetry

## Abstract

The cardiac autonomic nervous system (cANS) regulates cardiac adaptation to different demands. The heart is an asymmetrical organ, and in the selection of adequate treatment of cardiac diseases it may be relevant to take into account that the cANS also has sidedness as well as regional differences in anatomical, functional, and molecular characteristics. The left and right ventricles respond differently to adrenergic stimulation. Isoforms of nitric oxide synthase, which plays an important role in parasympathetic function, are also distributed asymmetrically across the heart. Treatment of cardiac disease heavily relies on affecting left-sided heart targets which are thought to apply to the right ventricle as well. Functional studies of the right ventricle have often been neglected. In addition, many principles have only been investigated in animals and not in humans. Anatomical and functional heterogeneity of the cANS in human tissue or subjects is highly valuable for understanding left- and right-sided cardiac pathology and for identifying novel treatment targets and modalities. Within this perspective, we aim to provide an overview and synthesis of anatomical and functional heterogeneity of the cANS in tissue or subjects, focusing on the human heart.

## Introduction

The cardiac autonomic nervous system (cANS) adapts the responses of the heart to external demands. It consists of a sympathetic part, which adapts cardiac function to physical activity and stress, and a parasympathetic part, which adapts it to a resting and restorative state. Under physiological conditions these systems are balanced and cardiac responses will be fine-tuned to differentiating demands. Under pathophysiological conditions, dysregulation of the cANS can occur as a result of for example cardiac damage and/or failure, and consequently the balance between the sympathetic and parasympathetic activity is lost. Usually, this is caused by an increased activity of the sympathetic part of the cANS and/or diminished activity of the parasympathetic part and is associated with an adverse prognosis.

Knowledge about the cANS is necessary for understanding cardiac disease and for the identification of treatment targets (Vegh et al., 2016). Much knowledge is derived from studies in patients with left-sided pathology, such as myocardial infarction or left ventricular failure (Cao et al., 2000; Fallavollita et al., 2014; Fukuda et al., 2015; Matsuo et al., 2016; Franciosi et al., 2017). The same principles are considered to apply to the right ventricle (RV). However, the RV is developmentally, morphologically, and functionally different from the left ventricle (LV). Traditional heart failure medication used in patients with left ventricular failure, relying heavily on influencing the cANS, may not have the same effect on patients with congenital heart disease and right ventricular failure, even though the hemodynamic problem is similar (Hechter et al., 2001; Lester et al., 2001; Robinson et al., 2002; Dore et al., 2005; Josephson et al., 2006; Doughan et al., 2007; Giardini et al., 2007; Therrien et al., 2008; Bouallal et al., 2010; Tutarel et al., 2012; Dos et al., 2013; van der Bom et al., 2013; Palma and Benarroch, 2014; Tobler et al., 2017). Likewise, other cardiac diseases show a relation to cardiac sidedness and region and are heavily influenced by autonomic innervation, such as cardiac arrhythmias originating from the region of the pulmonary veins, ligament of Marshall and the RV outflow tract (Wickramasinghe and Patel, 2013).

Although several reports describe the human heart and cANS, a detailed overview focusing specifically on anatomical sidedness and regional differences in cardiac autonomic innervation as related to function, is currently lacking. The selection of adequate treatment, the identification of future treatment targets, the planning of cardiothoracic surgery and catheter ablation procedures for arrhythmias, as well as the use of thoracic epidural anesthesia might be optimized by taking the sidedness and regional differences of the cANS into consideration.

The aim of the current paper is to review the anatomy and physiology of the human cANS with special emphasizes on asymmetry and regional differences in peripheral cardiac autonomic regulation. For a comprehensive overview of central regulation of cardiac autonomic function, we refer to previous excellent work (Anderson et al., 2000; Kirby, 2007; Kawashima, 2011; Hasan, 2013; Palma and Benarroch, 2014; Jamali et al., 2016; Coote and Spyer, 2018). Data from animal studies are cited when human data are unavailable or when data from animal studies offer additional insight. Relevant questions for future research are formulated.

## Sympathetic Ganglia and Nerves: Anatomical Evidence of Origins, Variations and Asymmetry (Figure 1A)

**FIGURE 1:**
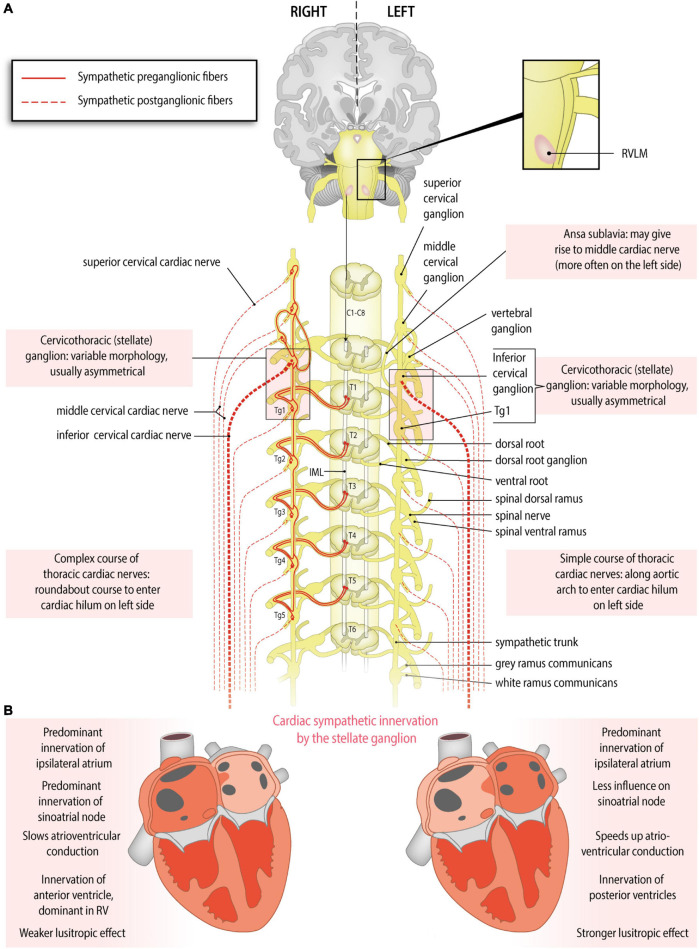
Anatomy of the sympathetic cardiac autonomic nervous system: asymmetry and regional differences. **(A)** Sympathetic cardiac autonomic nervous system. Preganglionic cardiac sympathetic axons (red, solid lines) arise from neurons of the intermediolateral (IML) cell columns in the upper four or five thoracic segments of the spinal cord. These neurons receive excitatory input from the rostral ventrolateral medulla (RVLM). The preganglionic fibers leave the spinal cord through ventral (anterior) roots, enter the ventral (anterior) rami of spinal nerves and pass to the sympathetic chain through white rami communicantes to synapse in the upper thoracic ganglia (Tg) or cervical ganglia; postganglionic fibers (red, dotted lines) from these ganglia form the sympathetic cardiac nerves. At the heart parasympathetic and sympathetic nerves converge to form the cardiac plexus from which atrial and ventricular autonomic innervation is arranged. Sided and regional differences in anatomy are indicated in the boxes. **(B)** Functional anatomy of cardiac sympathetic innervation by the right and left stellate ganglia. Sided and regional differences in function are indicated in the boxes. The right stellate ganglion greatly increases heart rate, slows the atrioventricular conduction, influences the right atrium more strongly than the left, shortens the QT interval, contributes to some extent to myocardial relaxation, and is the predominant source of sympathetic innervation in the right ventricle and the anterior part of both ventricles. The left stellate ganglion increases heart rate to some extent, speeds up the atrioventricular conduction, influences the left atrium more strongly than the right, lengthens the QT interval, contributes greatly to myocardial relaxation, and is the predominant source of sympathetic innervation of the posterior part of the ventricles.

Preganglionic cardiac sympathetic fibers originate from neurons located in the intermediolateral cell columns of the upper thoracic spinal cord (usually T1-T4 or T5) and exit the spinal cord through the ventral (anterior) roots of spinal nerves (Figure 1A). Subsequently, they synapse on postganglionic sympathetic neurons in the paravertebral ganglia of the sympathetic trunk. The sympathetic trunk is a bilateral structure situated left and right paravertebrally, extending from the cervical to the coccygeal level. The following ganglia of the sympathetic trunk may provide postganglionic fibers to the heart: the left and right superior cervical, middle cervical and vertebral ganglia, the left and right cervicothoracic ganglion (also called stellate ganglion, composed of the inferior cervical ganglion and the first thoracic ganglion), and the upper thoracic ganglia on both sides. However, there still is debate about the upper and lower limits from which postganglionic fibers to the heart originate, and significant interindividual variations exist (Bonica, 1968; Janes et al., 1986; Kawashima, 2011) (reviewed in Wink et al., 2020). Postganglionic sympathetic fibers travel as cardiac nerves from these ganglia toward the cardiac plexus. Defined by the paravertebral ganglion they originate from, these nerves are named as follows (Figure 1A): the superior cervical cardiac nerve (originating from the superior cervical ganglion or sympathetic trunk between the superior cervical and middle cervical ganglia), the middle cervical cardiac nerve (originating from the middle cervical ganglion or the vertebral ganglion, or from the sympathetic trunk between the middle cervical and the inferior cervical or cervicothoracic/stellate ganglia, including the ansa subclavia), the inferior cervical cardiac nerve (originating from the inferior cervical ganglion or the cervicothoracic/stellate ganglion), or thoracic cardiac nerves (originating from the thoracic ganglia or the thoracic sympathetic trunk below the inferior cervical or cervicothoracic/stellate ganglion) (Kawashima, 2005; Federative International Programme for Anatomical Terminology, 2019)

Variations and asymmetry in stellate ganglion morphology and cardiac sympathetic nerve origin have been described. In an American human cadaver study, it was observed that the right stellate ganglion was often longer than the left (Kwon et al., 2018). However, in a cohort from Switzerland, the left stellate ganglion was longer (Marcer et al., 2012) and in a Chinese cohort, no differences in length were found between the left and right stellate ganglion (Yin et al., 2015). A study of Pather in humans in South Africa, also reported no significant difference in the length and width of the right and left sides of the adult cardiothoracic ganglia (Pather et al., 2006). In these studies, differing occurrence and asymmetry of the left and right stellate ganglion as well as the other thoracic sympathetic ganglia is consistently described.

A middle cardiac cervical nerve originating from the ansa subclavia (nerve connection between the middle and inferior cervical ganglia) was observed twice as often on the left compared to the right (Kawashima, 2005). Furthermore, asymmetry in the origin and courses of the left and right thoracic cardiac nerves originating from the lower thoracic ganglia (fourth or fifth) was observed (Fukuyama, 1982; Kawashima, 2005). The left lower thoracic cardiac nerves follow a simple course along the aortic arch and thoracic aorta, comparable with most other cardiac nerves, which generally run along the great arteries. In contrast, the right lower thoracic cardiac nerves may descend obliquely along the intercostal vessels, turn and ascend along the thoracic aorta, to finally reach the heart through the right venous part of the cardiac hilum or by connecting to the cardiac plexus. This complex, ‘roundabout’ course may be related to remodeling/regression of the right aortic arch during embryonic development, whereas the left sided arch persists (Gittenberger-de Groot et al., 2006; Kawashima, 2011).

## Cardiac Areas of Sympathetic Innervation by the Left and Right Stellate Ganglion: Functional Evidence (Figure 1B and Table 1)

The stellate ganglia play important and differing roles in cardiac autonomic function and have been studied extensively. The right stellate ganglion is primarily responsible for increasing the heart rate and slowing atrioventricular conduction, whereas the left stellate ganglion has little effect on heart rate and increases atrioventricular conduction (Figure 1B). This was concluded from functional studies in humans. A right stellate ganglion block leads to a marked decrease in heart rate, whereas left stellate ganglion block leads to a more discrete decrease in heart rate (Rogers et al., 1978; Yokota et al., 2013). Furthermore, it was demonstrated that right stellate ganglion block leads to faster atrioventricular conduction and left stellate ganglion block leads to slower atrioventricular conduction (Cinca et al., 1985). Interestingly, a dog study suggested that the left stellate ganglion dominates over the right in terms of ventricular refractoriness: left stellate ganglion block led to a net increase in refractoriness, suggesting a decreased sensitivity to ventricular arrhythmias. However, while right stellate ganglion block produced a similar effect in absence of a functional left stellate ganglion, right stellate ganglion block led to a net decrease in refractoriness in the presence of a functional left stellate ganglion. This suggests an overshoot in compensatory sympathetic activity from the left stellate ganglion (Schwartz et al., 1977). Similarly, in humans, left stellate ganglion block shortens the corrected QT interval, whereas right stellate ganglion block lengthens it, suggesting a potential factor in arrhythmogenesis (Egawa et al., 2001). In line with this, left stellate ganglion block is increasingly used to treat ventricular arrhythmias (Meng et al., 2017). Other studies elaborate further on this topic (Lane and Schwartz, 1987; Schwartz et al., 1992; Zhou et al., 2008).

**TABLE 1:** Summary of functional characteristics of the right and left sided ganglion stellatum.^a^

Right stellate ganglion	Left stellate ganglion
Predominant innervation ipsilateral atrium ++	Predominant innervation ipsilateral atrium ++
Innervation contralateral atrium +	Innervation contralateral atrium +
Predominant innervation of anterior part both ventricles (dogs)	Predominant innervation of posterior part both ventricles (dogs)
Echocardiographic radial and circumferential strain in the anterior regions of the LV ↑ (pigs)	Echocardiographic radial and circumferential strain in the inferior/posterior regions of the LV ↑ (pigs)
Predominant innervation of RV (dogs)	
Myocardial relaxation (lusitropy) + (dogs)	Myocardial relaxation (lusitropy) ++ (dogs)
Myocardial contractility +	Myocardial contractility ++
**Cardiac conduction system**	
Heart rate ↑↑	Heart rate ↑/=
AV conduction ↓	AV conduction ↑
QT interval ↓	QT interval ↑
**Right stellate ganglion block**	**Left stellate ganglion block**
Heart rate ↓↓	Heart rate ↓
AV conduction ↑	AV conduction ↓
QTc interval ↑	QTc interval ↓
Atrial refractory time ↑	Atrial refractory time ↑
AF inducibility ↓	AF inducibility ↓

*^*a*^Data derived from human studies, unless otherwise indicated-for references see text.*

A block of either stellate ganglion in humans, led to prolonged atrial refractory time and a reduction in inducibility of atrial fibrillation. Each stellate ganglion may predominantly innervate the ipsilateral atrium, which, in turn, relays signals to the contralateral atrium (Leftheriotis et al., 2016).

The right stellate ganglion and left stellate ganglion influence both ventricles but unevenly. These regional differences were firstly demonstrated in dogs: the right stellate ganglion primarily influenced the anterior part of the LV and RV, whereas the left stellate ganglion primarily influenced the posterior LV and RV (Yanowitz et al., 1966). Similar to human studies, in this study QT-prolongation was observed after ablation of the right stellate ganglion and after stimulation of the left stellate ganglion (Yanowitz et al., 1966). A similar pattern was found in pigs: stimulation of the right stellate ganglion led to increased echocardiographic radial and circumferential strain in the anterior regions, whereas stimulation of the left stellate ganglion led to increased strain in the inferior/posterior regions (Zhou et al., 2013). This pattern was confirmed by measuring activation-recovery intervals in another pig study (Vaseghi et al., 2013). Additionally, a study by Schlack and Thamer (1996) in dogs demonstrated an improved lusitropic (myocardial relaxation) effect by left stellate ganglion stimulation compared with an impaired relaxation upon right stellate ganglion stimulation. These authors also demonstrated a higher global contractility increase with left stellate ganglion stimulation compared with right stellate ganglion stimulation. In the RV, innervation of the right stellate ganglion may predominate: in a study in dogs, right stellate ganglion stimulation shortened the refractory period more strongly in the RV than in the LV. Left stellate ganglion stimulation shortened the refractory period of the LV and the RV equally (Garcia-Calvo et al., 1992). Whether the same patterns in the ventricles are present in humans, and whether the distribution of innervation is influenced by the dominance of the coronary system, is unknown. It is considered that these differences in effects from the left and right stellate ganglia may play a role in arrhythmias, especially when their activity is unbalanced (Lane and Schwartz, 1987).

## Parasympathetic Innervation: Anatomical Evidence of Origins and Contributions From Left and Right Vagal Cardiac Branches (Figure 2A)

**FIGURE 2:**
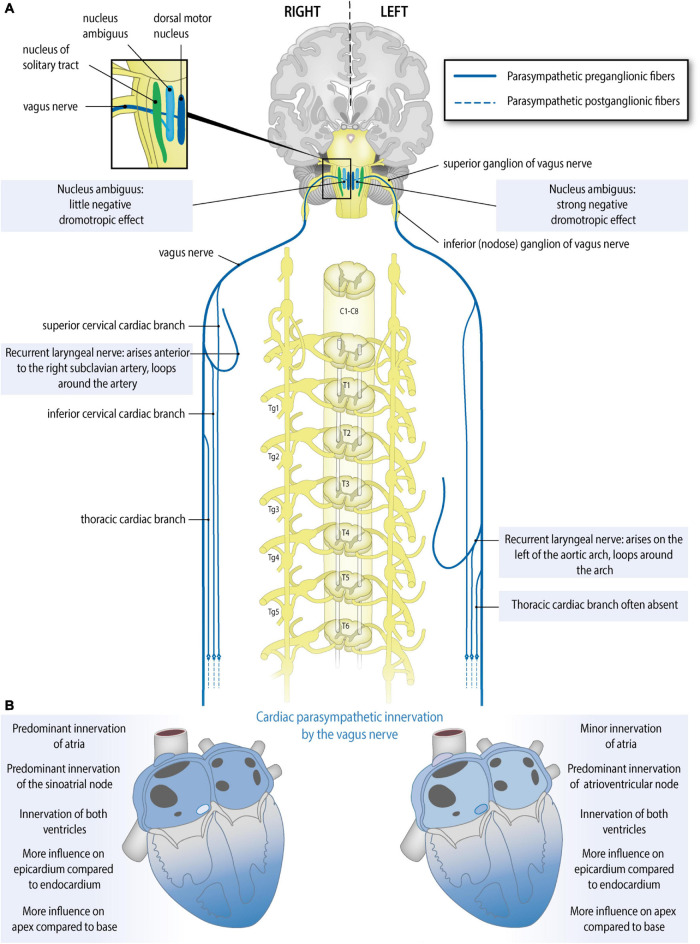
Anatomy of the parasympathetic cardiac autonomic nervous system: asymmetry and regional differences. **(A)** Parasympathetic cardiac autonomic nervous system. Preganglionic cardiac parasympathetic axons (blue, solid lines) arise from neurons in either the nucleus ambiguus or dorsal vagal nucleus; they run in cardiac branches of the vagus nerve to synapse in cardiac plexuses and ganglia from where postganglionic fibers (blue, dotted lines) innervate the sinoatrial node (SAN), atrioventricular node (AVN), coronary arteries, and ventricular myocytes. Sided and regional differences in anatomy are indicated in the boxes. **(B)** Functional anatomy of the right and left vagal nerves. Sided and regional differences in function are indicated in the boxes. The right vagus nerve greatly slows heart rate, may influence the atria more than the left vagus nerve, slows atrioventricular conduction to some extent, influences the epicardium more strongly than the endocardium and influences the apex more strongly than the base. The left vagus nerve slows heart rate to some extent, may influence the atria less than the right vagus nerve, greatly slows atrioventricular conduction, influences the epicardium more strongly than the endocardium and influences the apex more strongly than the base.

Preganglionic cardiac parasympathetic fibers originate from neurons located in the nucleus ambiguus and the dorsal motor nucleus of the vagus nerve and reach the heart through cardiac branches of this nerve (Standring and Gray, 2016). The vagus nerves (tenth cranial nerve) originate bilaterally from the medulla oblongata and give rise to a recurrent laryngeal nerve that differs in origin (branching site) and course on the two sides. Parasympathetic cardiac branches from these nerves are defined according to their origin as follows (Figure 2A): the superior cervical cardiac branch originates from the vagus nerve proximal to the branching site of the recurrent laryngeal nerve. A branch originating from any part of the recurrent laryngeal nerve is called an inferior cervical cardiac branch, and the cardiac branch originating from the vagus nerve distal to the branching site of the recurrent laryngeal nerve is called a thoracic cardiac branch (Kawashima, 2005; Federative International Programme for Anatomical Terminology, 2019). While all cardiac branches are consistently seen on the right side, on the left side, the thoracic cardiac branch was absent in 45% of cases (Kawashima, 2005). In contrast to the preganglionic cardiac sympathetic fibers, which synapse on postganglionic neurons within ganglia of the sympathetic trunk (i.e., remote from the heart), preganglionic cardiac parasympathetic fibers synapse on postganglionic neurons within ganglionated plexuses embedded in the epicardial fat pads and the heart wall. To our knowledge, no further information is available about variations and asymmetry in the parasympathetic cardiac branches.

## Cardiac Areas of Parasympathetic Innervation by the Left and Right Vagus Nerve: Functional Evidence (Figure 2B and Table 2)

The human sinoatrial node and atria are most likely predominantly influenced by the right vagus nerve, the atrioventricular node is predominantly influenced by the left vagus nerve, and the ventricles are influenced at least by the left vagus nerve but likely by both vagus nerves (Banzett et al., 1999; Lewis et al., 2001; Muppidi et al., 2011; Figure 2B). Although human studies are scarce, animal studies are in accordance with this: in the isolated rabbit heart, stimulation of the right vagus nerve had a stronger effect on heart rate, whereas the left vagus nerve had a stronger effect on atrioventricular conduction (Ng et al., 2001). This group also used the same model to explore this in the context of so-called accentuated antagonism (generally, vagal stimulation has a stronger effect when a background level of sympathetic activity is present). During sympathetic stimulation, right and left vagal nerve stimulation may reduce the heart rate differently: there was a trend toward a stronger effect of the right vagus nerve, although this result was not statistically significant (Brack et al., 2004). Animal research also reveals possible regional differences: in pigs, left or right vagus nerve stimulation had a stronger effect on the endocardium than on the epicardium and also on the apex compared to the base. This study also demonstrates that in pigs at least the LV is influenced by both vagus nerves (Yamakawa et al., 2014).

**TABLE 2:** Summary of functional characteristics of the right and left sided vagus nerves.^a^

Right vagus nerve	Left vagus nerve
Innervation atria ++?	Innervation atria +?
Sino-atrial node	AV node
Both ventricles	Both ventricles
Influence on endocardium > influence epicardium (pig)	Influence on endocardium > influence epicardium (pig)
Influence on apex > influence on base (pig)	Influence on apex > influence on base (pig)
**Cardiac conduction system**	
Heart rate ↓↓ (rabbit)	Heart rate ↓
AV conduction ↓	AV conduction ↓↓

*^*a*^Data derived from human studies, unless otherwise indicated-for references see text.*

## Cardiac Afferent Nerve Fibers: Asymmetry and Regional Differences, Anatomical and Functional Aspects

Although visceral afferent fibers, in a strict sense, are not part of the autonomic nervous system (Armour, 1999), given their clinical relevance, regional and asymmetrical features of the afferent (sensory) part of the cardiac nervous system are also considered here. Cardiac afferent nerve fibers are an integral part of the regulatory pathways the cANS is involved in, and transfer sensory signals from the heart to the central nervous system, where they may activate efferent neurons through feedback loops. They convey cardiac nociceptive and reflexive information (Armour, 1999; Kirby, 2007). Like cardiac efferent (autonomic) nerves, these afferents demonstrate regional differences and asymmetry. Studies conducted in humans are rare and most knowledge regarding the organization and function of the cardiac afferent system is derived and extrapolated from animal studies.

The neurites (dendrites) of cardiac afferent neurons are located in the myocardium and their cell bodies lie in the dorsal root ganglia of spinal nerves or the inferior ganglion of the vagus nerve (nodose ganglion) (Anderson et al., 2000; Kirby, 2007; Palma and Benarroch, 2014).

Cardiac spinal or “sympathetic” afferents [named as such because their fibers accompany sympathetic efferent (autonomic) fibers retrogradely in splanchnic nerves] convey mainly nociceptive sensory information from the heart via the splanchnic nerves, sympathetic trunk, spinal nerve and dorsal root (dorsal root ganglia) to the posterior horn of the spinal grey matter, where they synapse on second-order neurons of lamina I. The nociceptive information is further conveyed via ascending pain pathways to the thalamus and other brain regions involved in cardiac pain perception (Palma and Benarroch, 2014). When spinal cardiac afferents are involved in feedback loops via local interneurons projecting to the intermediolateral cell columns of the spinal cord, they can influence sympathetic efferent (motor) activity directly (Armour, 1999; Anderson et al., 2000; Kirby, 2007; Palma and Benarroch, 2014).

Some sympathetic afferents (mainly C-fibers, which are unmyelinated and are slow-conducting) produce substance P as their neurotransmitter. Substance P mediates nociception and also has efferent effects. Nerves containing substance P in the human heart are especially found around coronary arteries and other small blood vessels, and inside intrinsic cardiac ganglia (Weihe et al., 1981; Rechardt et al., 1986; Laine et al., 2000; Hoover et al., 2009). Substance P may be upregulated under pathological conditions. In conditions of ischemia-reperfusion, substance P can have a beneficial effect through increasing coronary blood flow (which was demonstrated in dogs). Conversely, in non-ischemic conditions such as myocarditis and volume overload, substance P may contribute to inflammation, apoptosis, and long-term reduction of LV-function (as was shown in various rodent models) (Dehlin and Levick, 2014).

Cardiac vagal afferents, of which unmyelinated, slow-conducting C-fibers constitute an important part, as was shown in studies in amongst others dogs and rats (Ditting et al., 2005) run centrally in the vagus nerves and convey mainly reflexive (mechano- and chemosensory) information from the heart via the nodose ganglion to the (caudal part of the) solitary nucleus (nucleus tractus solitarii) (Figure 2A). They can activate feedback loops through the thalamus and the parabrachial nucleus, and nucleus ambiguus, which lead to increased parasympathetic or decreased sympathetic outflow to the heart (Armour, 1999; Kirby, 2007; Palma and Benarroch, 2014). Of interest, cardiac afferent fibers have also been described to interact directly with postganglionic sympathetic neurons in the stellate ganglia and with parasympathetic postganglionic neurons or interneurons in the intrinsic cardiac ganglia, forming part of intrathoracic feedback loops, bypassing the central nervous system (Crick et al., 2000; Kirby, 2007).

The distribution of cardiac afferent nerve fibers in human can differ per cardiac region. The often observed bradycardia specifically accompanying inferoposterior myocardial infarctions may be a consequence of activation of vagal afferents on the inferior wall of the LV which trigger parasympathetic reflexes (Perez-Gomez et al., 1979; Flapan et al., 1993). The same pattern was observed in an experimental study in dogs (Thames et al., 1978). Interestingly, patients with an anterior myocardial infarction also had worse baroreflex sensitivity and heart rate variability at follow-up compared with patients with inferior/posterior infarctions, which may also be due to this distribution, although it must be noted that the left ventricular ejection fraction was also lower in the anterior infarction group (La Rovere et al., 1998).

Distribution of vagal afferents in the heart was studied in guinea pigs by retrograde labeling of the nodose ganglia. Most vagal afferents were concentrated in the posterior atrial wall (mostly on the ipsilateral atrium of the labeled nodose ganglion), the pulmonary arterial wall, and around the coronary arteries (Quigg et al., 1988). From a physiological study in cats, it was concluded that cardiac afferents (vagal or spinal not specified) were located throughout the heart wall. In the ventricles, they were present mostly in the endocardium. In the atria, they were present equally endo- and epicardially (Malliani et al., 1973).

In conclusion, the anatomy and physiology of cardiac afferents are still largely to be elucidated, especially in humans.

## Asymmetry of the Cardiac Plexus and Coronary Cardiac Nerves: Anatomical Evidence (Figure 3)

**FIGURE 3:**
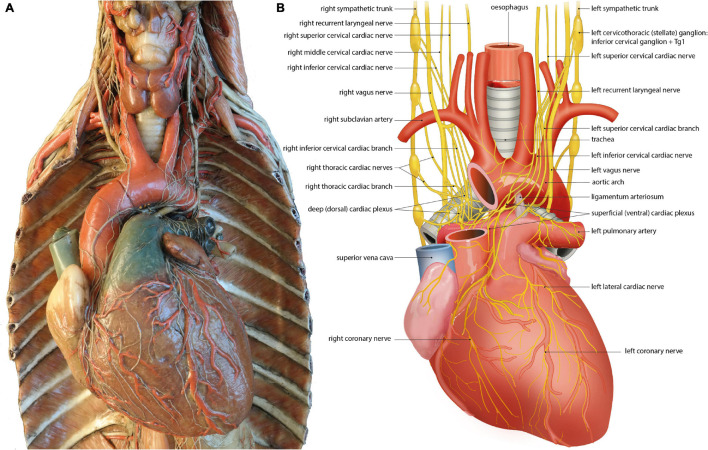
The left and right cardiac nerves and superficial and deep cardiac plexus. **(A)** Wax mold (Museo delle Cere Anatomiche “Luigi Cattaneo”, Bologna, Italy) demonstrating sympathetic and parasympathetic (vagal) cardiac nerves and superficial and deep cardiac plexus. Photomicrograph courtesy of Dr. E.A.J.F. Lakke. With permission of the Museo delle Cere Anatomiche “Luigi Cattaneo”, University Museum System, Alma Mater Studiorum – University of Bologna, Italy. **(B)** Annotated drawing, details depiction of cardiac nerves and plexus depicted in the left panel. At the heart sympathetic and parasympathetic nerves converge to form the superficial and deep cardiac plexus from which atrial and ventricular autonomic innervation is arranged. For further explanation see text.

Human left and right sympathetic and parasympathetic cardiopulmonary nerves connect in the mediastinum, where they form the cardiac plexus. A distinction is made between the superficial (ventral) cardiac plexus, located in between the pulmonary trunk and aortic arch, and the deep (dorsal) cardiac plexus, located between the aorta and trachea (De Gama et al., 2012; Figure 3). The superficial and deep cardiac plexuses are not as discrete and confined as for example the cervical and thoracic paravertebral ganglia, but rather describe the locations of interconnecting nerve networks where the number of nerve fibers gradually increases and nerve fibers tend to be more mixed (including sympathetic, parasympathetic and visceral afferent fibers). Plexus formation tends to start higher on the right side (level of the brachiocephalic trunk) than on the left side (level of the aortic arch) (Kawashima, 2011; Figure 3; Table 3). Possibly, this can be attributed to regional differences during embryonic development, as the initially symmetrical pharyngeal arch arterial system, giving rise to part of the putative arterial vasculature, will show a left-sided dominance, with disappearance of the right sixth pharyngeal arch artery and disappearance/remodeling of the right aortic arch artery. The right fourth pharyngeal arch artery will form the proximal part of the right subclavian artery, below which the right laryngeal recurrent nerve will eventually course (Gittenberger-de Groot et al., 2006).

**TABLE 3:** Summary of asymmetrical and regional features of the extrinsic cardiac plexus, coronary cardiac nerves, and intrinsic cardiac plexus (all human studies).

• Right-sided extrinsic cardiac plexus formation located more superiorly (level of brachiocephalic trunk) than left-sided extrinsic cardiac plexus (level of aortic arch) {Kawashima, 2011 #222}
• Right coronary cardiac nerve: composed largely from contributions of left stellate ganglion {Janes et al., 1986, #35}
• Left coronary cardiac nerve: composed largely from contributions of right stellate ganglion {Janes et al., 1986, #35}
• Dorsal and ventral right atrial intrinsic cardiac plexuses: mainly parasympathetic {Petraitiene et al., 2014 #410}
• Left and right coronary epicardiac plexuses: mainly sympathetic {Petraitiene et al., 2014, #410}
• Predominance of parasympathetic neurons in plexuses on the atria {Wake and Brack, 2016, #167}
• Predominance of sympathetic neurons in plexuses on the ventricles {Wake and Brack, 2016, #167}

Among many smaller nerves, three large mixed nerves arise from these plexuses that will innervate the atria and ventricles: from the deep/dorsal cardiac plexus, the left coronary cardiac nerve (which runs along the left anterior descending coronary artery) and the left lateral cardiac nerve (which runs along the circumflex coronary artery) arise. From the ventral plexus, the right coronary cardiac nerve (which runs along the right coronary artery) arises (Janes et al., 1986). Additional cardiopulmonary nerves connect to these (coronary) cardiac nerves distal from the plexuses. Surprisingly, the left coronary cardiac nerve is composed mainly of contributions of right-sided cardiac nerves, largely originating from the right stellate ganglion, which pass through the deep (dorsal) cardiac plexus. The right coronary cardiac nerve is composed mainly of contributions of left-sided cardiac nerves, largely originating from the left stellate ganglion, which pass through the superficial (ventral) cardiac plexus (Janes et al., 1986; Table 3).

## Intrinsic Cardiac Ganglia: Anatomical Evidence of Regional Organization and Gradient in (Para)Sympathetic Dominance (Figure 4)

Apart from nerves that conduct signals from the central nervous system to the human heart (i.e., extrinsic cardiac nerves), an intrinsic cardiac nervous system is also present. A network of over 800 ganglia can be found on the posterior surfaces of the atria, around the base of the aorta and pulmonary artery, dorsal and ventral to the pulmonary veins, and on the ventricular myocardium near the coronary arteries (Singh et al., 1996; Armour et al., 1997; Pauza et al., 2000; Wake and Brack, 2016). These ganglia are usually embedded in epicardial adipose tissue and are more or less organized into seven regions, called ganglionated (sub)plexuses: the right dorsal atrial plexus, the ventral right atrial plexus, the left dorsal plexus, the ventral left atrial plexus, the middle dorsal plexus, the right coronary plexus, and the left coronary plexus (Pauza et al., 2000; Wake and Brack, 2016). Nerve fibers in these plexuses are sympathetic, parasympathetic, or mixed. The dorsal and ventral right atrial ganglionated subplexuses (supplying also the sinoatrial node) are predominantly parasympathetic. The left and right coronary epicardiac subplexuses (supplying mostly the ventricles) are predominantly sympathetic (Petraitiene et al., 2014; Table 3). There is a predominance of parasympathetic neurons in plexuses on the atria and sympathetic neurons in plexuses on the ventricles (Wake and Brack, 2016). A considerable part of the intrinsic cardiac ganglion cells show co-expression of sympathetic and parasympathetic markers, as was shown in rhesus monkeys as well as humans (Weihe et al., 2005). This network has a function in passing down vagal impulses further and also in integrating sympathetic, parasympathetic, and sensory information in complex cardiac responses (Armour et al., 1997; Hasan, 2013; Wake and Brack, 2016).

**FIGURE 4:**
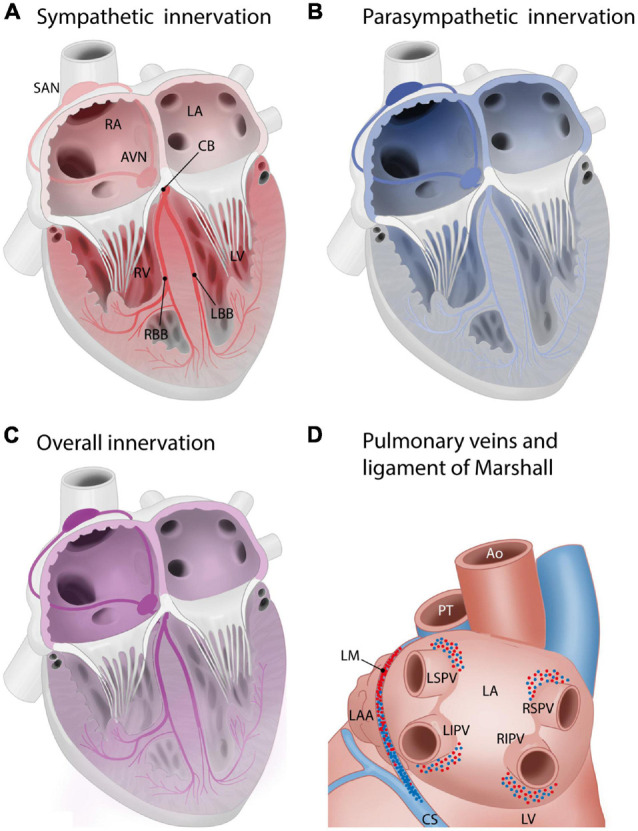
Innervation of the cardiac muscle, including the cardiac conduction system. Red: sympathetic innervation, blue: parasympathetic innervation. **(A,B)** Schematic overview of sympathetic **(A)** and parasympathetic **(B)** innervation of the cardiac compartments and cardiac conduction system. Innervation gradient ranging from the most to least dense innervation: sinoatrial node > atrioventricular node > penetrating bundles > bundle branches. The conduction system is more densely innervated than surrounding myocardium. Sinoatrial node and atrioventricular node: predominantly parasympathetic innervation, penetrating bundle and the bundle branches: predominantly sympathetic (Crick et al., 1994; Chow et al., 2001). **(C)** Global innervation densities in cardiac areas. Innervation density in the atria > innervation density in the ventricles. Gradient in innervation density (higher to lower) from base to the apex. Atria: most nerve endings parasympathetic. Ventricles: most nerve endings sympathetic. Sympathetic innervation density right atrium > left atrium. Right atrium and right ventricle: higher density of parasympathetic innervation than left atrium and left ventricle (Kawano et al., 2003). **(D)** Innervation of the pulmonary veins and ligament of Marshall, posterior view of the left atrium. Sympathetic and parasympathetic innervation density: highest above superior pulmonary veins and below inferior pulmonary veins (Tan et al., 2006). The ligament of Marshall is densely sympathetically and parasympathetically innervated (Han et al., 2010; Rodríguez-Mañero et al., 2016). From superior to inferior, it shifts from predominantly sympathetic to predominantly parasympathetic. AVN, atrioventricular node; AVB, atrioventricular/His bundle; CB, common bundle; LA, left atrium; LBB, left bundle branch; LIPV, left inferior pulmonary vein; LSPV, left superior pulmonary vein; LV, left ventricle; SAN, sinoatrial node; RA, right atrium; RBB, right bundle branch; RIPV, right inferior pulmonary vein; RSPV, right superior pulmonary vein; RV, right ventricle.

Interestingly, in the experimental treatment of refractory vagally mediated reflex syncope, ablation of both left- and right atrial sites of parasympathetic innervation has shown promising results. Even though an anatomical substrate for these conditions may be difficult to locate, it appears that damaging parasympathetic control of the heart on either side may prevent recurring episodes (Pachón et al., 2005; Sun et al., 2016).

## Anatomical Evidence of Regional Differences in Cardiac Innervation Density: Sympathetic and Parasympathetic

Generally, in humans, innervation density is higher in the atria than in the ventricles, and a gradient in innervation density (higher to lower) is present from the ventricular base to the apex (Kawano et al., 2003). In the atria, most nerve endings are parasympathetic, and in the ventricles, most nerve endings are sympathetic (Kawano et al., 2003). The right atrium (RA) has a higher density of sympathetic innervation than the left atrium (LA) (Kawano et al., 2003). The RA and RV have a higher density of parasympathetic innervation than the LA and LV (Kawano et al., 2003; Figure 4).

Specifically in the human conduction system, there is an innervation gradient ranging from the most dense innervation in the sinoatrial node to less innervation in the atrioventricular node, penetrating bundles, and bundle branches (Crick et al., 1994; Chow et al., 2001). As a whole, the conduction system is more densely innervated than the atrial and ventricular myocardium (Crick et al., 1994; Chow et al., 2001). The normal adult conduction system is innervated by both sympathetic and parasympathetic nerve branches (Crick et al., 1994; Chow et al., 2001). The sinoatrial node and atrioventricular node predominantly show parasympathetic innervation, whereas the penetrating bundle and the bundle branches predominantly show sympathetic innervation (Crick et al., 1994; Chow et al., 2001; Figures 4A,B).

Interestingly, the innervation of the human conduction system changes considerably with age. In infants, more sympathetic nerves are present in all regions of the conduction system compared to parasympathetic nerves. During childhood and adulthood, the number of parasympathetic nerves increases to the extent that the levels of sympathetic and parasympathetic nerves are equalized. In the elderly, there is a decline in both types of nerves (Chow et al., 2001). These data implicate that the autonomic innervation of the human cardiac conduction system changes with age.

In the adult human LA, the area around the pulmonary veins is densely innervated (Figure 4D): many ganglionated plexuses with both sympathetic and parasympathetic fibers are found in this area. The right pulmonary veins are mostly innervated by the dorsal right atrial subplexus and the middle dorsal subplexus, whereas the left superior pulmonary vein is innervated by the left dorsal subplexus and the left inferior pulmonary vein is innervated by the left and middle dorsal subplexus (Vaitkevicius et al., 2008, 2009; Wickramasinghe and Patel, 2013). Sympathetic and parasympathetic innervation density are both especially high in the superior aspects of the superior pulmonary veins and the inferior aspects of the inferior pulmonary veins. Innervation density was also higher epicardially than endocardially (Tan et al., 2006). The pulmonary venous myocardium is infamous for its potential for arrhythmogenesis (Haïssaguerre et al., 1998), and autonomic innervation has been recognized as a potential modulating factor (Chen et al., 2014). Of interest, the potential for arrhythmogenicity appears to differ between different pulmonary veins, with more foci found in the superior pulmonary veins as compared to the inferior veins (Haïssaguerre et al., 1998; Chen et al., 1999). Transient autonomic dysfunction and neural injury has been described after catheter ablation of pulmonary vein for atrial fibrillation (Hsieh et al., 1999; Scherschel et al., 2019). The ligament of Marshall, a remnant of the left vena cava inferior that regresses during embryonic development, is surrounded by a dense network of both sympathetic and parasympathetic fibers. From superior to inferior, in humans, the innervation of the ligament of Marshall shifts from predominantly sympathetic (nerve density) to predominantly parasympathetic (presence of parasympathetic ganglia) (Makino et al., 2006; Han et al., 2010; Rodríguez-Mañero et al., 2016; Figure 4D). The ligament of Marshall has nerve connections with the LA which are implicated in the genesis of atrial fibrillation (Han et al., 2010).

Animal studies reveal additional, potentially clinically relevant regional differences: in pigs, the RV was more densely innervated than the LV, whereas the left ventricular endocardium was more densely innervated than the right ventricular endocardium (Ulphani et al., 2010). In the RV outflow tract in dogs, sympathetic axons are found in the subendocardium as well as in the subepicardium, whereas in the remainder of the RV and the LV, sympathetic axons are only found in the subepicardium (Ito and Zipes, 1994), indicating a denser sympathetic innervation of the RV outflow tract as compared to the remainder of the RV. Whether this is also the case in humans is yet to be confirmed.

## Sidedness and Regional Differences in Cardiac Sympathetic Receptors and Responses: Anatomical and Functional Evidence

In preganglionic sympathetic nerve terminals, acetylcholine is the primary neurotransmitter. In postganglionic sympathetic nerve terminals, norepinephrine (NE) is the neurotransmitter primarily involved. Postganglionic sympathetic nerve fibers activate adrenergic receptors (the α1-, β1-, and β2-adrenergic receptors, of which the β-receptors greatly outnumber the α-receptors). β1-receptors, the predominant receptors in the heart, outnumber the β2-receptors in the atria and even more so in the ventricles. As the total β-receptor density is similar in the myocardial walls of all four cardiac chambers (Steinfath et al., 1992; Brodde et al., 2001), differences in the ratio β1 versus β2 receptors may be present between atria and ventricle or within the different cardiac compartments. Generally, stimulation of adrenergic receptors causes positive inotropy and an increase in heart rate. Though mostly present in the vascular wall, there are also α-adrenergic receptor in the ventricular myocardium, accounting for approximately 15% of the cardiac adrenergic receptors. Stimulation of the α1-myocardial receptors results in a weak positive inotropic response (Bristow et al., 1988; Table 4).

**TABLE 4:** Asymmetry and regional differences in the distribution and response of cardiac autonomic receptors and modulating factors.

Autonomic division	Receptor or modulating factor	Cardiac distribution	Asymmetrical/regional features of action
Sympathetic	Adrenergic	β1-receptors > β2-receptors in all chambers (human tissue)	Relative increase in inotropy after β-stimulation RV > LV (dogs)
		β-receptors > α-receptors in all chambers (human tissue)	Adrenergic stress may specifically lead to RVOT obstruction (dogs)
			α1-receptor stimulation: negative inotropy in RV, positive inotropy in LV (mice)
	NGF	NGF concentration ventricles > atria (rats)	
		NGF concentration LA > RA (rats)	
Parasympathetic	Muscarinic	M2-receptor concentration atria > ventricles (human tissue)	
		M2-receptor-specific tracer binding LV > RV > atria (human radioactive tracer imaging)	
	NOS	Expression eNOS left ventricular epicardium > RV > left ventricular endocardium (ferrets)	
		Expression nNOS left ventricular endocardium > left ventricular epicardium and RV (ferrets)	

*eNOS, endothelial nitric oxide synthase; LA, left atrium; LV, left ventricle; NGF, nerve growth factor; nNOS, neuronal nitric oxide synthase; NOS, nitric oxide synthase; RA, right atrium; RV, right ventricle.*

Linked to differences in recruitability of the systemic and pulmonary vasculature, the LV and the RV show differences in responses to adrenergic stimulation. In athletes, it was shown that during exercise, there is a greater relative increase of wall stress in the RV compared with the LV (La Gerche et al., 2011). In dogs receiving sympathetic stimulation, a stronger increase in systolic pressure was seen in the RV compared with the LV. This is likely mediated by β-receptors, as it was not affected by α-receptor blockade (Abe et al., 1987). A more recent study in dogs showed similar results: a stronger relative increase of contractility was seen in the RV compared with the LV after β-stimulation, which appeared to be related to interventricular differences in phosphodiesterase metabolism underlying the response to β-stimulation (Molina et al., 2014).

Adrenergic stress, for example treatment with inotropes, can cause dynamic RV outflow tract obstruction in humans, even without right ventricular hypertrophy (Denault et al., 2006). A study in pigs shows that the RV outflow tract indeed demonstrates an augmented response to adrenergic stimulation compared to the inflow tract, which was suggested by the authors as a possible mechanism to prevent excessive blood flow to the pulmonary circulation (Heerdt and Pleimann, 1996). This may be related to the additional presence of sympathetic axons in the deep myocardium specifically in the RV outflow tract, as was demonstrated in dogs (Ito and Zipes, 1994).

Furthermore, animal research shows that the RV and the LV may differ in their responses to sympathetic stimulation. *Ex vivo* stimulation of the α1-receptor with phenylephrine in ventricular trabeculae in mice resulted in negative inotropy in the RV and in positive inotropy in the LV (Wang et al., 2006).

Nerve growth factor (NGF), an important factor in nerve (re)growth which likely acts on the p75 neurotrophin receptor and tropomyosin-related receptor A (Li and Li, 2015), was investigated in rats of different ages (Saygili et al., 2012). Across all ages, nerve growth factor expression was higher in the ventricles than in the atria, and higher in the LA than in the RA. In the atria, nerve growth factor expression increased with age. In the ventricles, nerve growth factor expression was highest in neonatal rats. It decreased from neonatal to young age, to increase again at old age. These results may be related with an increase in sympathetic activation with age (Saygili et al., 2012). It is unknown whether these patterns can be extrapolated to humans. Regional differences in nerve growth factor expression in the human heart have not yet been investigated.

An important regulator of cardiac beta-adrenoreceptor signaling is the opioid receptor system. Three subtypes of endogenous opioids exist, all three of which have effects on the heart through their specific receptors: the μ opioid receptor, δ opioid receptor, and κ opioid receptor (Barron, 1999). The cardiac opioid system may especially be important as part of a negative feedback loop: conditions such as exercise and hypertension lead to increased cardiac opioid content, which depresses neurotransmitter release at adrenergic and/or vagal nerve terminals. Distribution of these receptors appears to be asymmetrical in rat studies: more δ opiate receptors are present on the right side of the heart compared to the left, and more receptors are furthermore present in the atria compared to the ventricles (Krumins et al., 1985). Activity of endogenous opioids (enkephalins), the ligands for these receptors, was generally higher in the atria than in the ventricles in a study in guinea pigs (Weihe et al., 1985).

The asymmetry and regional differences in the distribution and response of cardiac sympathetic receptors and modulating factors are summarized in Table 4.

## Asymmetry and Regional Differences in Cardiac Parasympathetic Receptors and Responses: Anatomical Evidence

Acetylcholine is the neurotransmitter employed in pre- and postganglionic parasympathetic nerve terminals. Postganglionic parasympathetic fibers mainly activate the muscarinic (M)2-cholinergic receptor. Stimulation of the M2-cholinergic receptor causes a negative inotropic effect and a decrease in heart rate (Brodde et al., 2001). An *in vitro* study in diseased human hearts showed a higher density of M2-receptors in the atria than in the ventricles (Deighton et al., 1990; Table 4). However, a radioactive tracer-imaging study in mainly healthy volunteers shows that the human LV and septum show the highest concentrations of the M2-receptor-specific tracer. Therefore, in physiological conditions the LV and septum may contain most of the physiologically active population of M2-receptors (Syrota et al., 1985; Table 4). In humans, the M2-receptor density decreases with age, as was shown by both functional evidence and *in vitro* study of the human RA (Brodde et al., 1998).

Nitric oxide (NO) plays an important role in parasympathetic regulation, as was demonstrated in animal models: under physiological circumstances, NO promotes acetylcholine release and reduces NE release from nerve terminals (Herring and Paterson, 2001; Dedkova Elena et al., 2003). Two different isoforms of nitric oxide synthase are primarily responsible for this: neuronal nitric oxide synthase (nNOS) in parasympathetic nerve terminals, and endothelial nitric oxide synthase (eNOS) in cardiac cells (Balligand et al., 2009).

From a clinical point of view, NO is implicated in vasovagal syncope: in young patients, this condition is mostly due to reduced systemic vascular resistance, which can be corrected by antagonizing NO (Stewart et al., 2017). In older patients, vasovagal syncope is mostly due to reduced cardiac output.

The distribution of NOS isoforms may be asymmetrical: in ferrets, eNOS expression is highest in the apical/midventricular epicardium of the LV, moderately high in the right ventricular free wall, and low in the left ventricular endocardium and the left ventricular side of the septum. In the sinoatrial node and the RA, eNOS is present in the majority of cells. The distribution of nNOS follows a more or less inverted pattern: the expression is high in the left ventricular endocardium and the left ventricular side of the septum, and low in the left ventricular epicardium and the RV (Brahmajothi and Campbell, 1999). The exact functional role and distribution of NOS isoforms in the human heart remains to be elucidated.

The asymmetry and regional differences in the distribution of cardiac parasympathetic receptors and modulating factors are summarized in Table 4.

## Summary and Clinical Implications

In the current review, we show that the human peripheral cANS shows considerable asymmetry, interindividual variations, and regional differences in anatomical, functional and molecular characteristics.

The right-sided lower thoracic cardiac nerves follow a complex course to reach the heart which differs greatly from the course of the left-sided lower thoracic cardiac nerves. The presence of the left-sided thoracic cardiac branch is highly variable, the localization of the cardiac plexus is higher on the right side compared to the left, and different parts of the cardiac plexus give rise to nerves innervating different parts of the heart. This is important to consider when planning thoracic surgery to avoid complications regarding autonomic function.

The left and right stellate ganglia and the left and right vagus nerves innervate different areas of the heart or have different effects on the same area. In particular, left stellate ganglion block may be used to treat ventricular arrhythmias. The RV outflow tract, pulmonary veins, and the ligament of Marshall have specific innervation gradients. Distribution of cardiac nerves and ganglia differs between regions, showing a parasympathetic predominance in the atria and a sympathetic predominance in the ventricles. The distribution of spinal and vagal afferent nerve fibers may differ between cardiac regions. The RV, the RV outflow tract, and the LV all respond differently to sympathetic stimulation. These factors heavily influence disease manifestation and efficacy of pharmaceutical treatment, and are therefore important to keep in mind. In addition, the risk of clinical procedures such as catheter ablation or stellate ganglion blockade for arrhythmias, may be reduced and their efficacy may be improved by taking asymmetry and regional differences of the cANS into consideration.

Many studies in this field are animal studies, and the RV is often neglected. Future research in human tissue or human subjects focusing for example on specific innervation of the RV outflow tract, distribution of cardiac afferents, regional differences in nerve growth factor or nitric oxide synthase expression, and differing effects of left- or right sided innervation on both the LV and the RV, would be highly valuable to comprehend the influence of cardiac innervation of disease course and potentially adjust treatments for specific cardiac diseases related to cardiac autonomic (dys)function.

## Author Contributions

TZ performed literature study, drafted the manuscript, co-designed the figures, and implemented suggestions by the co-authors. RN and MD wrote the parts of the manuscript, revised the manuscript and used literature extensively, co-designed the figures, and critically revised and approved of the final manuscript. JW wrote the parts of the manuscript, revised the manuscript and used literature extensively, and critically revised and approved of the final manuscript. PK, HV, and AE guided the process of drafting the manuscript and critically revised and approved of the final manuscript. MS critically revised and approved of the final manuscript. MJ conceived the concept of the manuscript, wrote parts of the manuscript, guided the literature study and the drafting of the manuscript, designed the figures, revised the manuscript and used literature extensively, and critically revised and approved of the final manuscript. All authors contributed to the article and approved the submitted version.

## Conflict of Interest

The authors declare that the research was conducted in the absence of any commercial or financial relationships that could be construed as a potential conflict of interest.

## Publisher’s Note

All claims expressed in this article are solely those of the authors and do not necessarily represent those of their affiliated organizations, or those of the publisher, the editors and the reviewers. Any product that may be evaluated in this article, or claim that may be made by its manufacturer, is not guaranteed or endorsed by the publisher.

## References

[B1] Abe Y. Saito D. Tani H. Nakatsu T. Kusachi S. Haraoka S. (1987). The effect of cardiac sympathetic nerve stimulation on the right ventricle in canine heart. *Jpn. Circ. J.* 51 535–542. 10.1253/jcj.51.535 3626013

[B2] Anderson R. Bandler R. Bohus B. Buijs R. Cechetto D. Clement C. (2000). *The Nervous System and The Heart*, ed. Ter Hors G. J. Totowa, NJ: Humana Press.

[B3] Armour J. A. (1999). Myocardial ischaemia and the cardiac nervous system. *Cardiovasc. Res.* 41 41–54. 10.1016/s0008-6363(98)00252-1 10325952

[B4] Armour J. A. Murphy D. A. Yuan B. X. Macdonald S. Hopkins D. A. (1997). Gross and microscopic anatomy of the human intrinsic cardiac nervous system. *Anat. Rec.* 247 289–298. 10.1002/(sici)1097-0185(199702)247:2<289::aid-ar15>3.0.co;2-l 9026008

[B5] Balligand J. L. Feron O. Dessy C. (2009). eNOS activation by physical forces: from short-term regulation of contraction to chronic remodeling of cardiovascular tissues. *Physiol. Rev.* 89 481–534. 10.1152/physrev.00042.2007 19342613

[B6] Banzett R. B. Guz A. Paydarfar D. Shea S. A. Schachter S. C. Lansing R. W. (1999). Cardiorespiratory variables and sensation during stimulation of the left vagus in patients with epilepsy. *Epilepsy Res.* 35 1–11. 10232789 10.1016/s0920-1211(98)00126-0

[B7] Barron B. A. (1999). Opioid peptides and the heart. *Cardiovasc. Res.* 43 13–16. 10536683 10.1016/s0008-6363(99)00112-1

[B8] Bonica J. J. (1968). Autonomic innervation of the viscera in relation to nerve block. *Anesthesiology* 29 793–813. 10.1097/00000542-196807000-00023 4874158

[B9] Bouallal R. Godart F. Francart C. Richard A. Foucher-Hossein C. Lions C. (2010). Interest of beta-blockers in patients with right ventricular systemic dysfunction. *Cardiol. Young* 20 615–619. 10.1017/s1047951110000764 20519056

[B10] Brack K. E. Coote J. H. Ng G. A. (2004). Interaction between direct sympathetic and vagus nerve stimulation on heart rate in the isolated rabbit heart. *Exp. Physiol.* 89 128–139. 10.1113/expphysiol.2003.002654 15109218

[B11] Brahmajothi M. V. Campbell D. L. (1999). Heterogeneous basal expression of nitric oxide synthase and superoxide dismutase isoforms in mammalian heart : implications for mechanisms governing indirect and direct nitric oxide-related effects. *Circ. Res.* 85 575–587. 10.1161/01.res.85.7.575 10506482

[B12] Bristow M. R. Minobe W. Rasmussen R. Hershberger R. E. Hoffman B. B. (1988). Alpha-1 adrenergic receptors in the nonfailing and failing human heart. *J. Pharmacol. Exp. Ther.* 247 1039–1045. 2849656

[B13] Brodde O. E. Bruck H. Leineweber K. Seyfarth T. (2001). Presence, distribution and physiological function of adrenergic and muscarinic receptor subtypes in the human heart. *Basic Res. Cardiol.* 96 528–538. 10.1007/s003950170003 11770070

[B14] Brodde O. E. Konschak U. Becker K. Rüter F. Poller U. Jakubetz J. (1998). Cardiac muscarinic receptors decrease with age. *In vitro* and *in vivo* studies. *J. Clin. Invest.* 101 471–478. 10.1172/jci1113 9435320 PMC508587

[B15] Cao J. M. Fishbein M. C. Han J. B. Lai W. W. Lai A. C. Wu T. J. (2000). Relationship between regional cardiac hyperinnervation and ventricular arrhythmia. *Circulation* 101 1960–1969. 10.1161/01.cir.101.16.1960 10779463

[B16] Chen P.-S. Chen L. S. Fishbein M. C. Lin S.-F. Nattel S. (2014). Role of the autonomic nervous System in Atrial Fibrillation: pathophysiology and therapy. *Circ. Res.* 114 1500–1515. 10.1161/circresaha.114.303772 24763467 PMC4043633

[B17] Chen S.-A. Hsieh M.-H. Tai C.-T. Tsai C.-F. Prakash V. S. Yu W.-C. (1999). Initiation of atrial fibrillation by ectopic beats originating from the pulmonary Veins: electrophysiological characteristics, pharmacological responses, and effects of radiofrequency ablation. *Circulation* 100 1879–1886. 10.1161/01.cir.100.18.1879 10545432

[B18] Chow L. T. Chow S. S. Anderson R. H. Gosling J. A. (2001). Autonomic innervation of the human cardiac conduction system: changes from infancy to senility–an immunohistochemical and histochemical analysis. *Anat. Rec.* 264 169–182. 10.1002/ar.1158 11590594

[B19] Cinca J. Evangelista A. Montoyo J. Barutell C. Figueras J. Valle V. (1985). Electrophysiologic effects of unilateral right and left stellate ganglion block on the human heart. *Am. Heart J.* 109 46–54. 10.1016/0002-8703(85)90414-4 3966332

[B20] Coote J. H. Spyer K. M. (2018). Central control of autonomic function. *Brain Neurosci. Adv.* 2:2398212818812012. 10.1177/2398212818812012 PMC7058216 32166159

[B21] Crick S. J. Sheppard M. N. Anderson R. H. (2000). “Nerve supply of the heart,” in *The Nervous System and the Heart*, ed. Ter Horst G. J. (Totowa, NJ: Humana), 3–54.

[B22] Crick S. J. Wharton J. Sheppard M. N. Royston D. Yacoub M. H. Anderson R. H. (1994). Innervation of the human cardiac conduction system. A quantitative immunohistochemical and histochemical study. *Circulation* 89 1697–1708. 7908612 10.1161/01.cir.89.4.1697

[B23] De Gama B. Z. Lazarus L. Partab P. Satyapai K. S. (2012). The sympathetic and parasympathetic contributions to the cardiac plexus: a fetal study. *Int. J. Morphol.* 30 1569–1576.

[B24] Dedkova Elena N. Ji X. Wang Yong G. Blatter Lothar A. Lipsius Stephen L. (2003). Signaling mechanisms that mediate nitric oxide production induced by acetylcholine exposure and withdrawal in cat atrial myocytes. *Circ. Res.* 93 1233–1240. 14615286 10.1161/01.RES.0000106133.92737.27

[B25] Dehlin H. M. Levick S. P. (2014). Substance P in heart failure: the good and the bad. *Int. J. Cardiol.* 170 270–277. 24286592 10.1016/j.ijcard.2013.11.010 PMC4450674

[B26] Deighton N. M. Motomura S. Borquez D. Zerkowski H. R. Doetsch N. Brodde O. E. (1990). Muscarinic cholinoceptors in the human heart: demonstration, subclassification, and distribution. *Naunyn Schmiedebergs Arch. Pharmacol.* 341 14–21. 2314479 10.1007/BF00195052

[B27] Denault A. Y. Chaput M. Couture P. Hebert Y. Haddad F. Tardif J. C. (2006). Dynamic right ventricular outflow tract obstruction in cardiac surgery. *J. Thorac. Cardiovasc. Surg.* 132 43–49. 16798301 10.1016/j.jtcvs.2006.03.014

[B28] Ditting T. Hilgers K. F. Scrogin K. E. Stetter A. Linz P. Veelken R. (2005). Mechanosensitive cardiac C-fiber response to changes in left ventricular filling, coronary perfusion pressure, hemorrhage, and volume expansion in rats. *Am. J. Physiol. Heart Circ. Physiol.* 288 H541–H552. 15471986 10.1152/ajpheart.00131.2004

[B29] Dore A. Houde C. Chan K. L. Ducharme A. Khairy P. Juneau M. (2005). Angiotensin receptor blockade and exercise capacity in adults with systemic right ventricles: a multicenter, randomized, placebo-controlled clinical trial. *Circulation* 112 2411–2416. 16216961 10.1161/CIRCULATIONAHA.105.543470

[B30] Dos L. Pujadas S. Estruch M. Mas A. Ferreira-Gonzalez I. Pijuan A. (2013). Eplerenone in systemic right ventricle: double blind randomized clinical trial. The evedes study. *Int. J. Cardiol.* 168 5167–5173. 23972966 10.1016/j.ijcard.2013.07.163

[B31] Doughan A. R. Mcconnell M. E. Book W. M. (2007). Effect of beta blockers (carvedilol or metoprolol XL) in patients with transposition of great arteries and dysfunction of the systemic right ventricle. *Am. J. Cardiol.* 99 704–706. 17317376 10.1016/j.amjcard.2006.10.025

[B32] Egawa H. Okuda Y. Kitajima T. Minami J. (2001). Assessment of QT interval and QT dispersion following stellate ganglion block using computerized measurements. *Reg. Anesth. Pain Med.* 26 539–544. 11707793 10.1053/rapm.2001.25935

[B33] Fallavollita J. A. Heavey B. M. Luisi A. J. Jr. Michalek S. M. Baldwa S. Mashtare T. L. Jr. (2014). Regional myocardial sympathetic denervation predicts the risk of sudden cardiac arrest in ischemic cardiomyopathy. *J. Am. Coll. Cardiol.* 63 141–149. 10.1016/j.jacc.2013.07.096 24076296 PMC3954563

[B34] Federative International Programme for Anatomical Terminology (2019). *Terminologia Anatomica.* Available online at: https://fipat.library.dal.ca/ (accessed January 9, 2021).

[B35] Flapan A. D. Wright R. A. Nolan J. Neilson J. M. Ewing D. J. (1993). Differing patterns of cardiac parasympathetic activity and their evolution in selected patients with a first myocardial infarction. *J. Am. Coll. Cardiol.* 21 926–931. 10.1016/0735-1097(93)90349-6 8450162

[B36] Franciosi S. Perry F. K. Roston T. M. Armstrong K. R. Claydon V. E. Sanatani S. (2017). The role of the autonomic nervous system in arrhythmias and sudden cardiac death. *Auton. Neurosci.* 205 1–11. 10.1016/j.autneu.2017.03.005 28392310

[B37] Fukuda K. Kanazawa H. Aizawa Y. Ardell J. L. Shivkumar K. (2015). Cardiac innervation and sudden cardiac death. *Circ. Res.* 116 2005–2019. 10.1161/CIRCRESAHA.116.304679 26044253 PMC4465108

[B38] Fukuyama U. (1982). Gross anatomy of the extrinsic cardiac nerve branches of human beings. *Acta Anat. Nippon* 57 357–380.6762795

[B39] Garcia-Calvo R. Chorro F. J. Sendra M. Alberola A. Sanchis J. Navarro J. (1992). The effects of selective stellate ganglion manipulation on ventricular refractoriness and excitability. *Pacing Clin. Electrophysiol.* 15 1492–1503. 10.1111/j.1540-8159.1992.tb02923.x 1383961

[B40] Giardini A. Lovato L. Donti A. Formigari R. Gargiulo G. Picchio F. M. (2007). A pilot study on the effects of carvedilol on right ventricular remodelling and exercise tolerance in patients with systemic right ventricle. *Int. J. Cardiol.* 114 241–246. 10.1016/j.ijcard.2006.01.048 21882492

[B41] Gittenberger-de Groot A. C. Azhar M. Molin D. G. M. (2006). Transforming growth factor β–SMAD2 signaling and aortic arch development. *Trends Cardiovasc. Med.* 16 1–6. 10.1016/j.tcm.2005.09.006 16387623

[B42] Haïssaguerre M. Jaïs P. Shah D. C. Takahashi A. Hocini M. Quiniou G. (1998). Spontaneous initiation of atrial fibrillation by ectopic beats originating in the pulmonary veins. *N. Engl. J. Med.* 339 659–666. 10.1056/NEJM199809033391003 9725923

[B43] Han S. Joung B. Scanavacca M. Sosa E. Chen P. S. Hwang C. (2010). Electrophysiological characteristics of the Marshall bundle in humans. *Heart Rhythm* 7 786–793. 10.1016/j.hrthm.2010.02.028 20188860 PMC2924610

[B44] Hasan W. (2013). Autonomic cardiac innervation: development and adult plasticity. *Organogenesis* 9 176–193. 10.4161/org.24892 23872607 PMC3896589

[B45] Hechter S. J. Fredriksen P. M. Liu P. Veldtman G. Merchant N. Freeman M. (2001). Angiotensin-converting enzyme inhibitors in adults after the Mustard procedure. *Am J Cardiol* 87 660–663.a611. 11230861 10.1016/s0002-9149(00)01452-1

[B46] Heerdt P. M. Pleimann B. E. (1996). The dose-dependent effects of halothane on right ventricular contraction pattern and regional inotropy in swine. *Anesth. Analg.* 82 1152–1158. 10.1213/00000539-199606000-00009 8638783

[B47] Herring N. Paterson D. (2001). Nitric oxide-cGMP pathway facilitates acetylcholine release and bradycardia during vagal nerve stimulation in the guinea-pig *in vitro*. *J. Physiol.* 535 507–518. 10.1111/j.1469-7793.2001.00507.x 11533140 PMC2278790

[B48] Hoover D. B. Isaacs E. R. Jacques F. Hoard J. L. Pagé P. Armour J. A. (2009). Localization of multiple neurotransmitters in surgically derived specimens of human atrial ganglia. *Neuroscience* 164 1170–1179. 10.1016/j.neuroscience.2009.09.001 19747529

[B49] Hsieh M.-H. Chiou C.-W. Wen Z.-C. Wu C.-H. Tai C.-T. Tsai C.-F. (1999). Alterations of heart rate variability after radiofrequency catheter ablation of focal atrial fibrillation originating from pulmonary veins. *Circulation* 100 2237–2243. 10.1161/01.CIR.100.22.2237 10577997

[B50] Ito M. Zipes D. P. (1994). Efferent sympathetic and vagal innervation of the canine right ventricle. *Circulation* 90 1459–1468. 10.1161/01.CIR.90.3.1459 8087953

[B51] Jamali H. K. Waqar F. Gerson M. C. (2016). Cardiac autonomic innervation. *J. Nucl. Cardiol.* 24 1558–1570. 10.1007/s12350-016-0725-7 27844333

[B52] Janes R. D. Brandys J. C. Hopkins D. A. Johnstone D. E. Murphy D. A. Armour J. A. (1986). Anatomy of human extrinsic cardiac nerves and ganglia. *Am. J. Cardiol.* 57 299–309. 10.1016/0002-9149(86)90908-2 3946219

[B53] Josephson C. B. Howlett J. G. Jackson S. D. Finley J. Kells C. M. (2006). A case series of systemic right ventricular dysfunction post atrial switch for simple D-transposition of the great arteries: the impact of beta-blockade. *Can. J. Cardiol.* 22 769–772. 10.1016/S0828-282X(06)70293-8 16835671 PMC2560517

[B54] Kawano H. Okada R. Yano K. (2003). Histological study on the distribution of autonomic nerves in the human heart. *Heart Vessels* 18 32–39. 12644879 10.1007/s003800300005

[B55] Kawashima T. (2005). The autonomic nervous system of the human heart with special reference to its origin, course, and peripheral distribution. *Anat. Embryol.* 209 425–438. 10.1007/s00429-005-0462-1 15887046

[B56] Kawashima T. (2011). Anatomy of the cardiac nervous system with clinical and comparative morphological implications. *Anat. Sci. Int.* 86 30–49. 10.1007/s12565-010-0096-0 21116884

[B57] Kirby M. L. (2007). *Cardiac Development.* Oxford: Oxford University Press.

[B58] Krumins S. A. Faden A. I. Feuerstein G. (1985). Opiate binding in rat hearts: modulation of binding after hemorrhagic shock. *Biochem. Biophys. Res. Commun.* 127 120–128. 10.1016/S0006-291X(85)80134-0 3977917

[B59] Kwon O. J. Pendekanti S. Fox J. N. Yanagawa J. Fishbein M. C. Shivkumar K. (2018). Morphological spectra of adult human stellate ganglia: implications for thoracic sympathetic denervation. *Anat. Rec. (Hoboken)* 301 1244–1250. 10.1002/ar.23797 29451372 PMC7105504

[B60] La Gerche A. Heidbuchel H. Burns A. T. Mooney D. J. Taylor A. J. Pfluger H. B. (2011). Disproportionate exercise load and remodeling of the athlete’s right ventricle. *Med. Sci. Sports Exerc.* 43 974–981. 21085033 10.1249/MSS.0b013e31820607a3

[B61] La Rovere M. T. Bigger J. T. Jr. Marcus F. I. Mortara A. Schwartz P. J. (1998). Baroreflex sensitivity and heart-rate variability in prediction of total cardiac mortality after myocardial infarction. ATRAMI (Autonomic Tone and Reflexes After Myocardial Infarction) Investigators. *Lancet* 351 478–484. 9482439 10.1016/s0140-6736(97)11144-8

[B62] Laine P. Naukkarinen A. Heikkilä L. Penttilä A. Kovanen P. T. (2000). Adventitial mast cells connect with sensory nerve fibers in atherosclerotic coronary arteries. *Circulation* 101 1665–1669. 10.1161/01.CIR.101.14.1665 10758048

[B63] Lane R. D. Schwartz G. E. (1987). Induction of lateralized sympathetic input to the heart by the CNS during emotional arousal: a possible neurophysiologic trigger of sudden cardiac death. *Psychosom. Med.* 49 274–284. 10.1097/00006842-198705000-00006 3602297

[B64] Leftheriotis D. Flevari P. Kossyvakis C. Katsaras D. Batistaki C. Arvaniti C. (2016). Acute effects of unilateral temporary stellate ganglion block on human atrial electrophysiological properties and atrial fibrillation inducibility. *Heart Rhythm* 13 2111–2117. 10.1016/j.hrthm.2016.06.025 27353237

[B65] Lester S. J. Mcelhinney D. B. Viloria E. Reddy G. P. Ryan E. Tworetzky W. (2001). Effects of losartan in patients with a systemically functioning morphologic right ventricle after atrial repair of transposition of the great arteries. *Am. J. Cardiol.* 88 1314–1316. 10.1016/S0002-9149(01)02098-7 11728365

[B66] Lewis M. E. Al-Khalidi A. H. Bonser R. S. Clutton-Brock T. Morton D. Paterson D. (2001). Vagus nerve stimulation decreases left ventricular contractility *in vivo* in the human and pig heart. *J. Physiol.* 534 547–552. 10.1111/j.1469-7793.2001.00547.x 11454971 PMC2278718

[B67] Li C. Y. Li Y. G. (2015). Cardiac sympathetic nerve sprouting and susceptibility to ventricular arrhythmias after myocardial infarction. *Cardiol. Res. Pract.* 2015:698368. 10.1155/2015/698368 26793403 PMC4697091

[B68] Makino M. Inoue S. Matsuyama T. A. Ogawa G. Sakai T. Kobayashi Y. (2006). Diverse myocardial extension and autonomic innervation on ligament of Marshall in humans. *J. Cardiovasc. Electrophysiol.* 17 594–599. 10.1111/j.1540-8167.2006.00375.x 16836704

[B69] Malliani A. Recordati G. Schwartz P. J. (1973). Nervous activity of afferent cardiac sympathetic fibres with atrial and ventricular endings. *J. Physiol.* 229 457–469. 10.1113/jphysiol.1973.sp010147 4724832 PMC1350316

[B70] Marcer N. Bergmann M. Klie A. Moor B. Djonov V. (2012). An anatomical investigation of the cervicothoracic ganglion. *Clin. Anat.* 25 444–451. 10.1002/ca.21266 22488995

[B71] Matsuo S. Nakajima K. Nakata T. (2016). Prognostic value of cardiac sympathetic nerve imaging using long-term follow-up data - ischemic vs. non-ischemic heart failure etiology. *Circ. J.* 80 435–441. 10.1253/circj.CJ-15-0952 26638869

[B72] Meng L. Tseng C. H. Shivkumar K. Ajijola O. (2017). Efficacy of stellate ganglion blockade in managing electrical storm: a systematic review. *JACC Clin. Electrophysiol.* 3 942–949. 10.1016/j.jacep.2017.06.006 29270467 PMC5734652

[B73] Molina C. E. Johnson D. M. Mehel H. Spatjens R. L. Mika D. Algalarrondo V. (2014). Interventricular differences in beta-adrenergic responses in the canine heart: role of phosphodiesterases. *J. Am. Heart. Assoc.* 3:e000858. 10.1161/JAHA.114.000858 24904016 PMC4309082

[B74] Muppidi S. Gupta P. K. Vernino S. (2011). Reversible right vagal neuropathy. *Neurology* 77 1577–1579. 10.1212/WNL.0b013e318233b3a2 21975204 PMC3198976

[B75] Ng G. A. Brack K. E. Coote J. H. (2001). Effects of direct sympathetic and vagus nerve stimulation on the physiology of the whole heart - a novel model of isolated langendorff perfused rabbit heart with intact dual autonomic innervation. *Exp. Physiol.* 86 319–329. 10.1113/eph8602146 11471534

[B76] Pachón J. C. Pachón E. Páchon J. C. Lobo T. Pachón M. Z. Vargas R. N. (2005). “Cardioneuroablation” – new treatment for neurocardiogenic syncope, functional AV block and sinus dysfunction using catheter RF-ablation. *EP Europace* 7 1–13. 10.1016/j.eupc.2004.10.003 15670960

[B77] Palma J. A. Benarroch E. E. (2014). Neural control of the heart: recent concepts and clinical correlations. *Neurology* 83 261–271. 10.1212/WNL.0000000000000605 24928126

[B78] Pather N. Partab P. Singh B. Satyapal K. S. (2006). Cervico-thoracic ganglion: its clinical implications. *Clin. Anat.* 19 323–326. 10.1002/ca.20214 16317739

[B79] Pauza D. H. Skripka V. Pauziene N. Stropus R. (2000). Morphology, distribution, and variability of the epicardiac neural ganglionated subplexuses in the human heart. *Anat. Rec.* 259 353–382. 10.1002/1097-0185(20000801)259:4<353::AID-AR10>3.0.CO;2-R 10903529

[B80] Perez-Gomez F. Martin De Dios R. Rey J. Garcia Aguado A. (1979). Prinzmetal’s angina:reflex cardiovascular response during episode of pain. *Br. Heart J.* 42 81–87. 10.1136/hrt.42.1.81 475938 PMC482116

[B81] Petraitiene V. Pauza D. H. Benetis R. (2014). Distribution of adrenergic and cholinergic nerve fibres within intrinsic nerves at the level of the human heart hilum. *Eur. J. Cardiothorac. Surg.* 45 1097–1105. 10.1093/ejcts/ezt575 24335471

[B82] Quigg M. Elfvin L.-G. Aldskogius H. (1988). Distribution of cardiac sympathetic afferent fibers in the guinea pig heart labeled by anterograde transport of wheat germ agglutinin-horseradish peroxidase. *J. Auton. Nerv. Syst.* 25 107–118. 10.1016/0165-1838(88)90015-X 3148648

[B83] Rechardt L. Aalto-Setälä K. Purjeranta M. Pelto-Huikko M. Kyösola K. (1986). Peptidergic innervation of human atrial myocardium: an electron microscopical and immunocytochemical study. *J. Auton. Nerv. Syst.* 17 21–32. 10.1016/0165-1838(86)90041-X 2430006

[B84] Robinson B. Heise C. T. Moore J. W. Anella J. Sokoloski M. Eshaghpour E. (2002). Afterload reduction therapy in patients following intraatrial baffle operation for transposition of the great arteries. *Pediatr. Cardiol.* 23 618–623. 10.1007/s00246-002-0046-2 12530495

[B85] Rodríguez-Mañero M. Schurmann P. Valderrábano M. (2016). Ligament and Vein of Marshall. A therapeutic opportunity in atrial fibrillation. *Heart Rhythm* 13 593–601. 10.1016/j.hrthm.2015.10.018 26576705 PMC4796610

[B86] Rogers M. C. Battit G. Mcpeek B. Todd D. (1978). Lateralization of sympathetic control of the human sinus node: ECG changes of stellate ganglion block. *Anesthesiology* 48 139–141. 10.1097/00000542-197802000-00009 655444

[B87] Saygili E. Kluttig R. Rana O. R. Saygili E. Gemein C. Zink M. D. (2012). Age-related regional differences in cardiac nerve growth factor expression. *Age (Dordr)* 34 659–667. 10.1007/s11357-011-9262-0 21559866 PMC3337926

[B88] Scherschel K. Hedenus K. Jungen C. Lemoine M. D. Rübsamen N. Veldkamp M. W. (2019). Cardiac glial cells release neurotrophic S100B upon catheter-based treatment of atrial fibrillation. *Sci. Transl. Med.* 11:eaav7770. 10.1126/scitranslmed.aav7770 31118294

[B89] Schlack W. Thamer V. (1996). Unilateral changes of sympathetic tone to the heart impair left ventricular function. *Acta Anaesthesiol. Scand.* 40 262–271. 10.1111/j.1399-6576.1996.tb04430.x 8848929

[B90] Schwartz P. J. Motolese M. Pollavini G. Lotto A. Ruberti U. G. O. Trazzi R. (1992). Prevention of sudden cardiac death after a first myocardial infarction by pharmacologic or surgical antiadrenergic interventions. *J. Cardiovasc. Electrophysiol.* 3 2–16. 10.1111/j.1540-8167.1992.tb01090.x

[B91] Schwartz P. J. Verrier R. L. Lown B. (1977). Effect of stellectomy and vagotomy on ventricular refractoriness in dogs. *Circ. Res.* 40 536–540. 870234 10.1161/01.res.40.6.536

[B92] Singh S. Johnson P. I. Lee R. E. Orfei E. Lonchyna V. A. Sullivan H. J. (1996). Topography of cardiac ganglia in the adult human heart. *J. Thorac. Cardiovasc. Surg.* 112 943–953. 10.1016/S0022-5223(96)70094-6 8873720

[B93] Standring S. Gray H. (2016). *Gray’s Anatomy : The Anatomical Basis of Clinical Practice.* Edinburgh: Churchill Livingstone.

[B94] Steinfath M. Lavicky J. Schmitz W. Scholz H. Doring V. Kalmar P. (1992). Regional distribution of beta 1- and beta 2-adrenoceptors in the failing and nonfailing human heart. *Eur. J. Clin. Pharmacol.* 42 607–611. 1320570 10.1007/BF00265923

[B95] Stewart J. M. Sutton R. Kothari M. L. Goetz A. M. Visintainer P. Medow M. S. (2017). Nitric oxide synthase inhibition restores orthostatic tolerance in young vasovagal syncope patients. *Heart* 103 1711–1718. 10.1136/heartjnl-2017-311161 28501796 PMC5819615

[B96] Sun W. Zheng L. Qiao Y. Shi R. Hou B. Wu L. (2016). Catheter ablation as a treatment for vasovagal syncope: long-term outcome of endocardial autonomic modification of the left atrium. *J. Am. Heart Assoc.* 5:e003471. 10.1161/JAHA.116.003471 27402231 PMC5015383

[B97] Syrota A. Comar D. Paillotin G. Davy J. M. Aumont M. C. Stulzaft O. (1985). Muscarinic cholinergic receptor in the human heart evidenced under physiological conditions by positron emission tomography. *Proc. Natl. Acad. Sci. U.S.A.* 82 584–588. 10.1073/pnas.82.2.584 3871527 PMC397085

[B98] Tan A. Y. Li H. Wachsmann-Hogiu S. Chen L. S. Chen P. S. Fishbein M. C. (2006). Autonomic innervation and segmental muscular disconnections at the human pulmonary vein-atrial junction: implications for catheter ablation of atrial-pulmonary vein junction. *J. Am. Coll. Cardiol.* 48 132–143. 10.1016/j.jacc.2006.02.054 16814659

[B99] Thames M. D. Klopfenstein H. S. Abboud F. M. Mark A. L. Walker J. L. (1978). Preferential distribution of inhibitory cardiac receptors with vagal afferents to the inferoposterior wall of the left ventricle activated during coronary occlusion in the dog. *Circ. Res.* 43 512–519. 10.1161/01.RES.43.4.512 688555

[B100] Therrien J. Provost Y. Harrison J. Connelly M. Kaemmerer H. Webb G. D. (2008). Effect of angiotensin receptor blockade on systemic right ventricular function and size: a small, randomized, placebo-controlled study. *Int. J. Cardiol.* 129 187–192. 10.1016/j.ijcard.2008.04.056 18672299

[B101] Tobler D. Bouchardy J. Reto E. Heg D. Muller C. Frenk A. (2017). Effect of phosphodiesterase-5 inhibition with Tadalafil on SystEmic Right VEntricular size and function - A multi-center, double-blind, randomized, placebo-controlled clinical trial - SERVE trial - Rational and design. *Int. J. Cardiol.* 243 354–359. 10.1016/j.ijcard.2017.05.079 28566262

[B102] Tutarel O. Meyer G. P. Bertram H. Wessel A. Schieffer B. Westhoff-Bleck M. (2012). Safety and efficiency of chronic ACE inhibition in symptomatic heart failure patients with a systemic right ventricle. *Int J Cardiol* 154 14–16. 10.1016/j.ijcard.2010.08.068 20843567

[B103] Ulphani J. S. Cain J. H. Inderyas F. Gordon D. Gikas P. V. Shade G. (2010). Quantitative analysis of parasympathetic innervation of the porcine heart. *Heart Rhythm* 7 1113–1119. 10.1016/j.hrthm.2010.03.043 20381645

[B104] Vaitkevicius R. Saburkina I. Rysevaite K. Vaitkeviciene I. Pauziene N. Zaliunas R. (2009). Nerve supply of the human pulmonary veins: an anatomical study. *Heart Rhythm* 6 221–228. 10.1016/j.hrthm.2008.10.027 19187915

[B105] Vaitkevicius R. Saburkina I. Zaliunas R. Pauziene N. Vaitkeviciene I. Schauerte P. (2008). Innervation of pulmonary veins: morphologic pattern and pathways of nerves in the human fetus. *Ann. Anat.* 190 158–166. 10.1016/j.aanat.2007.09.002 18413269

[B106] van der Bom T. Winter M. M. Bouma B. J. Groenink M. Vliegen H. W. Pieper P. G. (2013). Effect of valsartan on systemic right ventricular function: a double-blind, randomized, placebo-controlled pilot trial. *Circulation* 127 322–330. 10.1161/CIRCULATIONAHA.112.135392 23247302

[B107] Vaseghi M. Yamakawa K. Sinha A. So E. L. Zhou W. Ajijola O. A. (2013). Modulation of regional dispersion of repolarization and T-peak to T-end interval by the right and left stellate ganglia. *Am. J. Physiol. Heart Circ. Physiol.* 305 H1020–H1030. 10.1152/ajpheart.00056.2013 23893168 PMC3798747

[B108] Vegh A. M. D. Duim S. N. Smits A. M. Poelmann R. E. Ten Harkel A. D. J. Deruiter M. C. (2016). Part and parcel of the cardiac autonomic nerve system: unravelling its cellular building blocks during development. *J. Cardiovasc. Dev. Dis.* 3:28. 10.3390/jcdd3030028 29367572 PMC5715672

[B109] Wake E. Brack K. (2016). Characterization of the intrinsic cardiac nervous system. *Auton. Neurosci.* 199 3–16. 10.1016/j.autneu.2016.08.006 27568996

[B110] Wang G. Y. Mccloskey D. T. Turcato S. Swigart P. M. Simpson P. C. Baker A. J. (2006). Contrasting inotropic responses to alpha1-adrenergic receptor stimulation in left versus right ventricular myocardium. *Am. J. Physiol. Heart Circ. Physiol.* 291 H2013–H2017. 10.1152/ajpheart.00167.2006 16731650

[B111] Weihe E. Mcknight A. T. Corbett A. D. Kosterlitz H. W. (1985). Proenkephalin- and prodynorphin- derived opioid peptides in guinea-pig heart. *Neuropeptides* 5 453–456. 10.1016/0143-4179(85)90052-6 3839054

[B112] Weihe E. Reinecke M. Opherk D. Forssmann W. G. (1981). Peptidergic innervation (substance P) in the human heart. *J. Mol. Cell. Cardiol.* 13 331–333. 10.1016/0022-2828(81)90321-7 6167732

[B113] Weihe E. Schütz B. Hartschuh W. Anlauf M. Schäfer M. K. Eiden L. E. (2005). Coexpression of cholinergic and noradrenergic phenotypes in human and nonhuman autonomic nervous system. *J. Comp. Neurol.* 492 370–379. 10.1161/CIRCULATIONAHA.113.001596 16217790 PMC2593918

[B114] Wickramasinghe S. R. Patel V. V. (2013). Local innervation and atrial fibrillation. *Circulation* 128 1566–1575. 24081952 10.1161/CIRCULATIONAHA.113.001596 PMC3844530

[B115] Wink J. Van Delft R. Notenboom R. G. E. Wouters P. F. Deruiter M. C. Plevier J. W. M. (2020). Human adult cardiac autonomic innervation: controversies in anatomical knowledge and relevance for cardiac neuromodulation. *Auton. Neurosci.* 227:102674. 10.1016/j.autneu.2020.102674 32497872

[B116] Yamakawa K. So E. L. Rajendran P. S. Hoang J. D. Makkar N. Mahajan A. (2014). Electrophysiological effects of right and left vagal nerve stimulation on the ventricular myocardium. *Am. J. Physiol. Heart Circ. Physiol.* 307 H722–H731. 10.1152/ajpheart.00279.2014 25015962 PMC4187397

[B117] Yanowitz F. Preston J. B. Abildskov J. A. (1966). Functional distribution of right and left stellate innervation to the ventricles. Production of neurogenic electrocardiographic changes by unilateral alteration of sympathetic tone. *Circ. Res.* 18 416–428. 10.1161/01.RES.18.4.416 4952701

[B118] Yin Z. Yin J. Cai J. Sui T. Cao X. (2015). Neuroanatomy and clinical analysis of the cervical sympathetic trunk and longus colli. *J. Biomed. Res.* 29 501–507. 26668584 10.7555/JBR.29.20150047 PMC4662212

[B119] Yokota S. Taneyama C. Goto H. (2013). Different effects of right and left stellate ganglion block on systolic blood pressure and heart rate. *Open J. Anesth.* 3 143–147. 10.4236/ojanes.2013.33033

[B120] Zhou S. Jung B. C. Tan A. Y. Trang V. Q. Gholmieh G. Han S. W. (2008). Spontaneous stellate ganglion nerve activity and ventricular arrhythmia in a canine model of sudden death. *Heart Rhythm* 5 131–139. 10.1016/j.hrthm.2007.09.007 18055272

[B121] Zhou W. Yamakawa K. Benharash P. Ajijola O. Ennis D. Hadaya J. (2013). Effect of stellate ganglia stimulation on global and regional left ventricular function as assessed by speckle tracking echocardiography. *Am. J. Physiol. Heart Circ. Physiol.* 304 H840–H847. 10.1152/ajpheart.00695.2012 23335795 PMC3602776

